# Bleeding Risk in Elderly Patients Undergoing Percutaneous Coronary Intervention: A Comprehensive Review

**DOI:** 10.3390/jcm14041194

**Published:** 2025-02-12

**Authors:** Alexander Marschall, Fernando Rivero, David del Val, Teresa Bastante, Edurne López Soberón, Inés Gómez Sánchez, Elena Basabe Velasco, Fernando Alfonso, José María de la Torre Hernández, David Martí Sánchez

**Affiliations:** 1 Cardiology Department, Instituto de Investigación Sanitaria, Instituto de Investigación del Hospital de La Princesa (IIS-IP), Hospital Universitario de La Princesa, Universidad Autónoma de Madrid, 28006 Madrid, Spain; 2 Cardiology Department, Central Defense Hospital Gómez Ulla, University of Alcalá, 28801 Madrid, Spain; 3 Cardiology Department, Hospital Universitario Marqués de Valdecilla, IDIVAL, 39008 Santander, Spain

**Keywords:** bleeding risk, elderly, percutaneous coronary intervention, ARC-HBR, PRECISE-DAPT

## Abstract

The care of elderly patients with coronary artery disease (CAD) undergoing percutaneous coronary interventions (PCIs) presents unique challenges due to age-related physiological and functional changes. With the global population aging rapidly, this demographic change affects a growing proportion of individuals requiring PCI. However, advanced age is associated with increased susceptibility to ischemic and bleeding complications, driven by physiological changes such as altered coagulation, vascular stiffness, and declining organ function. These factors complicate the management of CAD, making the balance between reducing thrombotic events and minimizing bleeding risks particularly challenging. Antiplatelet therapy is central to post-PCI management, but its benefits and risks differ significantly in elderly patients compared to younger populations. Tools like the PRECISE-DAPT and ARC-HBR provide guidance on dual antiplatelet therapy duration and bleeding risk stratification. However, their applicability and predictive accuracy in elderly patients remain areas of active investigation. This underscores the need for improved risk assessment methods tailored to the unique needs of aging individuals. In this review, we explore the epidemiological, pathophysiological, and clinical aspects of CAD in elderly patients, emphasizing the impact of aging on disease presentation and outcomes. Furthermore, we assess current risk stratification tools and discuss their limitations in predicting adverse events in older populations. By synthesizing these insights, we aim to highlight the complexities of managing elderly CAD patients and identify opportunities for optimizing personalized care to achieve better outcomes in this vulnerable group.

## 1. Demographic Challenges of the 21st Century 

There is currently no universally accepted definition of an “elderly patient”, as chronological age alone may not adequately capture the diversity observed within this unique population [1]. However, many clinical practice guidelines categorize individuals aged ≥75 years as a high-risk population, and this age group is widely recognized as representative of the elderly [1,2]. Due to rising life expectancy, which is mainly driven by medical progress, the elderly are the most rapidly growing segment of the population in developed countries [3]. Advances in medicine have significantly contributed to rising life expectancy, making the elderly the fastest-growing segment of the population in developed nations. According to Eurostat, the proportion of people aged over 80 in Europe doubled between 2001 and 2020 [4]. Similarly, in the United States, the number of octogenarians increases by over 160,000 annually, with this group projected to grow fivefold by 2040 [2,5].

While the extension of life expectancy is a testament to medical and public health achievements, it has also given rise to a demographic characterized by age-related physiological and functional changes [1].

As a result, the elderly represent a particularly vulnerable patient subgroup, requiring heightened attention and tailored care from healthcare providers.

## 2. Pathophysiologic Changes in the Elderly

**Figure 1:**
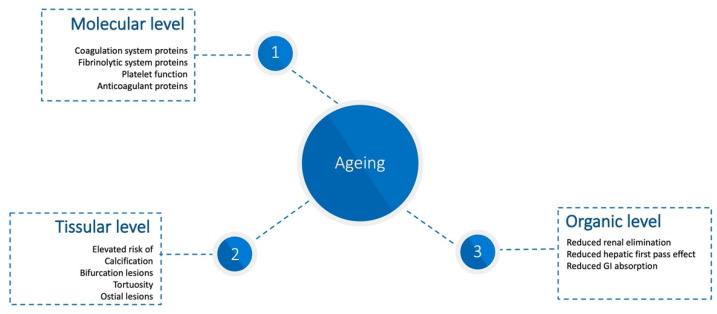
Pathophysiologic changes in the elderly with impacts on hemorrhagic and thrombotic risk.

Aging, though an inevitable process, is not a disease in itself. However, it is accompanied by a range of pathophysiological changes at the molecular, tissue, and organ levels [3]. These changes significantly influence the risks of both hemorrhagic and thrombotic events through complex interactions. (Figure 1). Consequently, older adults are at high risk for these events. For instance, in the Patient Related Outcomes with Endeavor versus Cypher Stenting Trial (PROTECT), age had an almost equal impact on bleeding and ischemic events (OR: 1.38; 95% CI 1.22–1.56 vs. OR: 1.37; 95% CI 1.25–1.50, respectively) [6].

### 2.1. Alterations at the Molecular Level

**Figure 2:**
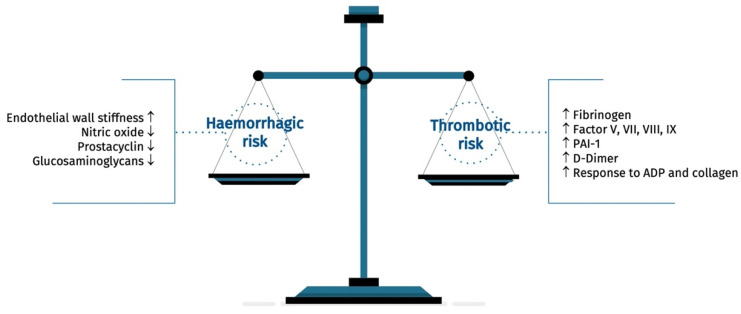
Changes in the equilibrium of hemostasis associated with aging.

The hemostatic system relies on a delicate equilibrium, involving interactions between coagulation proteins, fibrinolytic proteins, platelets, and the vascular endothelium [7]. Aging disrupts this balance, increasing the risks of both bleeding and clotting (Figure 2).

Key changes include coagulation system proteins [7,8,9,10], fibrinolytic proteins [11,12,13], platelet function [14], and the vascular endothelium [15].

### 2.2. Alterations at the Tissue Level

Aging induces structural changes in the vascular wall, including modifications to the extracellular matrix, vascular smooth muscle, and endothelium [7,16]. These changes heighten risks such as calcification, vessel tortuosity, and complex vascular injuries involving bifurcations and ostial lesions. Necropsy studies reveal a high prevalence of advanced coronary artery disease features in older adults, including [17,18,19].

### 2.3. Alterations at the Organic Level

Aging is also associated with functional declines in organs, which impact drug metabolism and pharmacokinetics. Key changes include reduced renal elimination, altered hepatic first-pass metabolism, and decreased gastrointestinal drug absorption [1,3].

## 3. Coronary Artery Disease in the Elderly 

### 3.1. Prevalence of Coronary Artery Disease in the Elderly

The pathophysiological changes associated with aging contribute to the high prevalence of coronary artery disease (CAD) among older adults. CAD is the leading cause of morbidity and mortality in this population, with age being the strongest risk factor for its development [20,21,22]. According to the National Health and Nutrition Examination Survey (NHANES), the prevalence of CAD rises steadily with age and displays a notable sex disparity—30.6% in men and 21.7% in women aged ≥80 years [20].

Additionally, a significant proportion of elderly individuals have subclinical CAD. Studies such as the Multi-Ethnic Study of Atherosclerosis (MESA) and the Cardiovascular Health Study (CHS) reveal subclinical CAD prevalence rates as high as 60–90% in patients aged ≥80 years [23,24].

### 3.2. Clinical Approach to Coronary Artery Disease in the Elderly 

**Figure 3:**
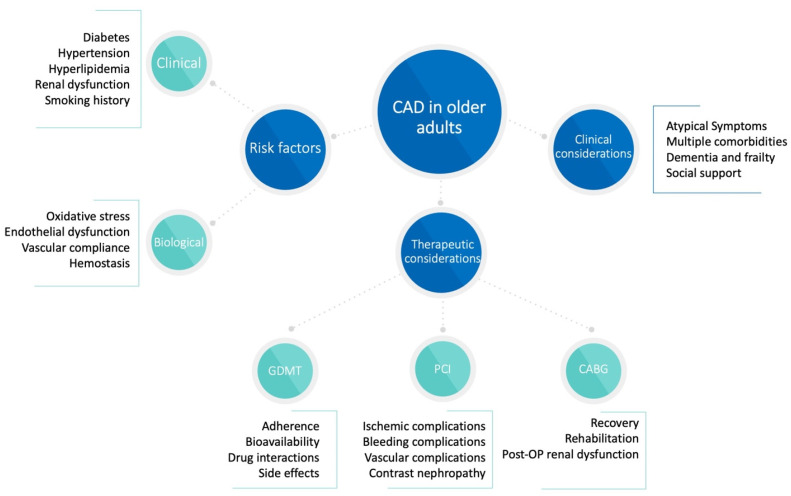
Clinical approach to CAD in the elderly.

Assessing CAD in elderly patients requires consideration of their unique characteristics, which can be categorized into the following [22] (Figure 3):

1. Risk factors;
2. Clinical considerations;
3. Therapeutic considerations.

Diagnosing CAD in older adults is particularly challenging due to the prevalence of atypical or nonspecific symptoms. Instead of presenting with classic angina, these patients may experience dyspnea, fatigue, nausea, or epigastric pain, which can complicate timely recognition and management [25,26]. Furthermore, age-related physiological changes significantly impact the pharmacokinetics and pharmacodynamics of medications [27,28].

## 4. Percutaneous Coronary Interventions in the Elderly 

### 4.1. Temporal Trends of PCI in Older Adults

The use of percutaneous coronary intervention (PCI) in older patients has steadily increased over time. Data from the prospective Acute Myocardial Infarction in Switzerland (AMIS) cohort, covering the years 2001 to 2012, revealed that PCI utilization in patients aged ≥70 years rose from 43.8% to 69.6% [29]. Notably, the most substantial growth occurred among the oldest cohorts, with a twofold increase in PCI use for patients aged ≥80 years and a threefold increase in those aged ≥90 years.

### 4.2. Outcomes of PCI in Older Adults

Despite the inherent challenges of managing older adults, who often present with higher rates of comorbidities and frailty, the outcomes of PCI in this demographic have improved. In-hospital mortality for elderly patients with acute coronary syndrome (ACS) decreased significantly, from 11.6% to 10.0%, alongside a reduction in in-hospital major adverse cardiac and cerebrovascular events (MACCEs) from 14.4% to 11.3%. Among octogenarians, there was a striking 22.7% relative decrease in mortality [29].

These findings align with previous research emphasizing the benefits of PCI in older populations. For instance, a pooled analysis of the FRISC II-ICTUS-RITA-3 (FIR) studies highlighted that a routine invasive strategy for ACS compared to a selective invasive approach resulted in lower risks of cardiovascular death and myocardial infarction in patients aged 65–74 years and ≥75 years but not in those <65 years [30].

The “After Eighty” trial further reinforced these observations. This study randomized patients aged ≥80 years with non-ST-elevation myocardial infarction (NSTEMI) or unstable angina pectoris to either an invasive or conservative strategy. The invasive group experienced a significant reduction in the composite primary outcome of myocardial infarction, urgent revascularization, stroke, and death (40.6% vs. 61.4%; HR 0.53, 95% CI 0.41–0.69, *p* = 0.0001) [31].

### 4.3. Considerations for Frail and Very Elderly Patients

While the benefits of PCI are well-documented in selected elderly patients, recent evidence underscores the importance of individualized care in frail and very elderly populations. For instance, the MOSCA-FRAIL study, focusing on frail patients with an average age of 86 years, highlighted the potential “costs” of routine invasive strategies, including a higher incidence of complications such as bleeding [32]. These findings emphasize the need for thorough risk prognostication and a personalized approach to treatment in this complex subgroup.

## 5. Impact of Hemorrhagic Complications After PCI

Outcomes for patients undergoing PCI have markedly improved with advances in antithrombotic therapy. However, the reduction in ischemic events is often accompanied by an increase in bleeding complications, which remain the most common non-cardiac adverse event following PCI. Bleeding is associated with a significant rise in mortality across the spectrum of CAD patients [33,34,35].

**Figure 4:**
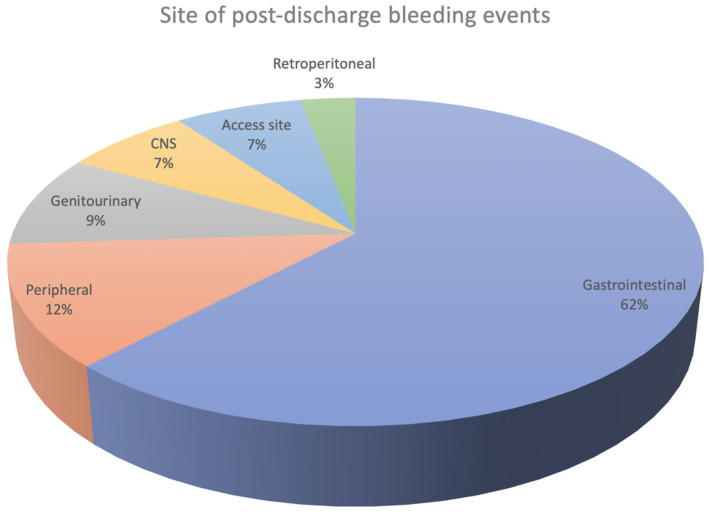
Site of post-discharge bleeding.

In the Assessment of Dual Anti-Platelet Therapy with Drug-Eluting Stents (ADAPT-DES) study, involving 8557 patients, 6.2% experienced bleeding events during long-term follow-up, with 15% of these patients reporting multiple events [35]. Among 444 patients with identified bleeding sources, the gastrointestinal tract emerged as the most common site of post-discharge hemorrhage (Figure 4). Importantly, hemorrhagic events during follow-up were strongly correlated with subsequent mortality, with adjusted hazard ratios even exceeding those for post-discharge myocardial infarction. This highlights the critical impact of bleeding complications on patient outcomes after PCI.

A meta-analysis of 40 studies, including data from 525,691 patients, corroborated these findings, showing a significantly higher risk of mortality among patients who experienced bleeding (5.8%) compared to those without major bleeding events (2.4%) [34].

## 6. Bleeding Risk of the Elderly Population

Despite the promising outcomes of PCI in older patients, as highlighted previously, bleeding events remain the most frequent complications in this group and are particularly concerning in the elderly. Hemorrhagic complications are significantly more prevalent in older adults compared to younger patients [36].

This heightened risk can be attributed to several factors, including a higher prevalence of comorbid conditions such as chronic kidney disease (CKD), arterial hypertension, and more advanced atherosclerosis, alongside the molecular and tissue-level changes associated with aging [37].

The impact of blood loss is especially severe in older patients due to its potential to cause hypovolemia, hypotension, reduced oxygen-carrying capacity, drug discontinuation, and the need for blood transfusions. These consequences collectively exacerbate the detrimental effects of bleeding events in this vulnerable population [33,38,39].

### 6.1. Definition of Bleeding Events

Major bleeding rates after PCI vary widely in the published literature, influenced by several factors, one of which is the specific definition used for hemorrhagic events [40]. Historically, one of the most commonly used bleeding scales was the Thrombolysis in Myocardial Infarction (TIMI) criteria, developed to classify bleeding events following thrombolysis for ST segment elevation myocardial infarction (STEMI) [41]. While the TIMI definition has evolved over time to include a broader range of bleeding scenarios, it remains focused primarily on identifying acute and severe bleeding events. Similarly, the Global Utilization of Streptokinase and Tissue Plasminogen Activator for Occluded Coronary Arteries (GUSTO) criteria are designed to identify only the most critical bleeding episodes [42]. The most recent BARC consensus statement was specifically developed to provide a more standardized approach to bleeding definitions. It offers a comprehensive classification system with five major categories of bleeding [43,44].

Table 1 provides a summary of the most commonly applied bleeding definitions.

### 6.2. Bleeding Risk and Antiplatelet Therapy in Coronary Artery Disease

For the past three decades, dual antiplatelet therapy (DAPT) has been the standard of care for patients undergoing PCI [26,47,48,49]. However, the optimal duration of DAPT remains a subject of ongoing debate, and various studies have examined different DAPT regimens [50,51,52,53,54,55,56].

Table 2 summarizes one-year bleeding rates in trials of antiplatelet therapy after coronary stenting.

According to clinical practice guidelines, the first and most crucial step in determining the appropriate duration of DAPT after PCI is assessing the individual patient’s bleeding risk [26,66,67]. Several trials suggest that shortening DAPT duration may be considered for patients with low ischemic risk [50,68,69,70].

Conversely, based on the findings from the 12 or 30 months of dual antiplatelet therapy after drug-eluting stents (DAPT) and the long-term use of ticagrelor in patients with prior myocardial infarction (PEGASUS-TIMI 54) trials, extending DAPT beyond 12 months should be considered for patients with high ischemic risk provided they are not at high risk of bleeding [53,55].

## 7. Prediction of Bleeding Events

Généreux et al. analyzed data from the ADAPT-DES study to identify predictors of major bleeding events after PCI in a general population. Several factors were found to be associated with increased bleeding risk, including advanced age, use of oral anticoagulants at discharge, peripheral artery disease, calcified lesions, bifurcation lesions, platelet reactivity, and baseline hemoglobin levels [35]. To enhance bleeding risk stratification, several studies have proposed risk scores [71,72,73], with the ESC guidelines recommending the use of the PRECISE-DAPT score for this purpose. This score incorporates age, previous bleeding history, white blood cell count, baseline hemoglobin, and creatinine clearance to predict bleeding risk within a 12-month period [73]. See Table 3 for details. Validation studies have demonstrated that the PRECISE-DAPT score is effective, with a C-index ranging from 0.65 to 0.71 in multiple cohorts. A score of ≥25 indicates a high bleeding risk, and in these cases, the guidelines recommend shortening the duration of dual antiplatelet therapy (DAPT) to 3 months [67,74].

The 2023 ESC guidelines also introduced the ARC-HBR (Academic Research Consortium for High Bleeding Risk) criteria to streamline bleeding risk assessment. While this tool is intended to classify patients into high- or low-risk categories, it is relatively complex, consisting of 20 clinical criteria divided into major and minor categories, making it difficult to use in routine clinical practice [67,75]. Furthermore, a subgroup analysis from the SWEDEHEART study showed that the PRECISE-DAPT score’s predictive ability declines in elderly patients, especially those with pre-existing bleeding risk factors such as advanced age [76]. In this study, the C-statistic for the PRECISE-DAPT score dropped from 0.64 in the general population to 0.57 in older adults. Similarly, a smaller study of patients aged ≥75 years found that most elderly individuals had PRECISE-DAPT scores above the recommended cut-off of ≥25, limiting the utility of this score in this cohort [77].

In our retrospective multicenter study, we evaluated the performance of both the PRECISE-DAPT and ARC-HBR scores in an exclusively elderly population [78]. Our findings confirmed that the PRECISE-DAPT score had modest discriminatory ability (C-statistic of 0.601), consistent with the SWEDEHEART study. A higher cut-off value for this score did not significantly improve its predictive accuracy. Moreover, this study was the first to assess the ARC-HBR criteria in an elderly cohort, and we found that it did not adequately predict bleeding risk in this group. Interestingly, applying a higher cut-off value of ≥2 points (with minor criteria assigned 0.5 points and major criteria one point) for the ARC-HBR criteria significantly improved its predictive value, contrasting with the PRECISE-DAPT score, which places more emphasis on age.

**Figure 5:**
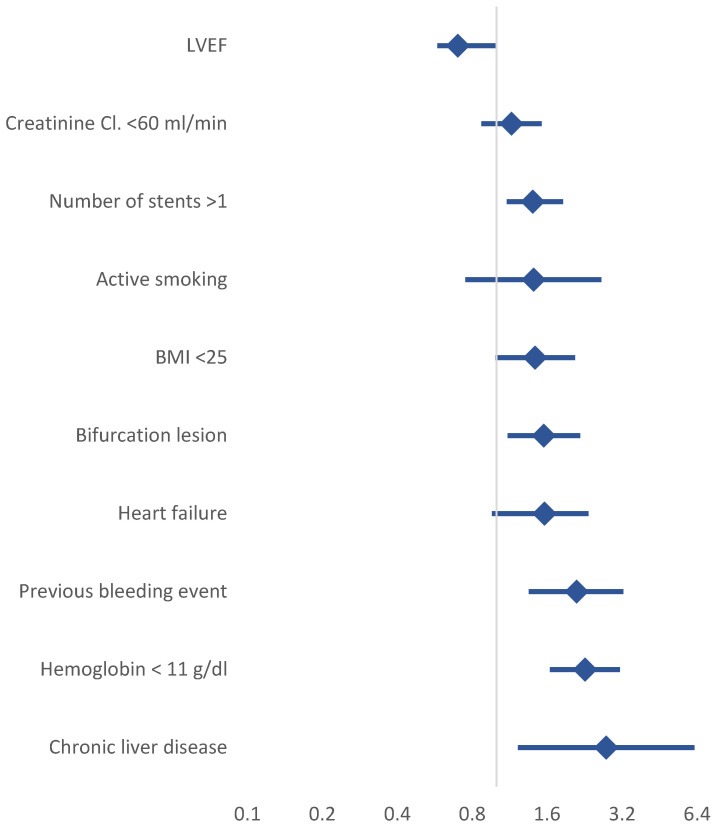
Adjusted HR for prediction of bleeding events.

Finally, we developed a simplified clinical evaluation score (SCE) to predict bleeding risk in elderly patients. Our results showed that an SCE ≥ 1 significantly predicted post-discharge bleeding (PDB) at 12 months and outperformed both the PRECISE-DAPT and ARC-HBR scores. The SCE demonstrated comparable discriminatory power to these established tools, suggesting that it may be a useful alternative in clinical practice for elderly patients, particularly in specific clinical scenarios (Figure 5).

## 8. Prediction of MACCEs and Clinical Impact of the Interbalance Between MACCEs and Bleeding

### 8.1. Risk Stratification in PCI

While risk scores like the EuroSCORE II are well-established tools for predicting major adverse cardiac and cerebrovascular events (MACCEs) in surgical revascularization, PCI risk stratification remains less standardized. One widely accepted PCI scoring system is the SYNTAX score, originally developed from the SYNTAX trial, which focuses primarily on anatomical features [79]. The subsequent SYNTAX II score incorporates patient-related parameters and has demonstrated superiority over its predecessor, highlighting the value of including clinical variables in PCI risk assessment [80]. However, both SYNTAX scores are primarily intended to guide decision-making between coronary artery bypass grafting (CABG) and PCI.

A novel approach, the CHIP-PCI score, was recently introduced based on an analysis of the UK BCIS Database (2006–2016). This score incorporates 12 risk factors (seven patient-related and five procedure-related) to assess the complexity of PCI procedures. Unlike the SYNTAX scores, CHIP-PCI focuses on balancing the risks and benefits of PCI for individual patients [81,82]. However, the original study population had a relatively young mean age, with even the high-risk CHIP subgroup averaging below 75 years (66 and 71 years, respectively). Additionally, the primary endpoint was in-hospital MACCEs, defined as a composite of death, periprocedural stroke, or periprocedural myocardial infarction.

Table 4 summarizes the 13 independent risk factors for in-hospital MACCEs in these patients.

### 8.2. Evaluation of CHIP-PCI in Elderly Populations

To assess the utility of the CHIP-PCI score in elderly patients, we conducted a retrospective multicenter study that included 2725 patients with a mean age of 81 ± 4 years. Among the cohort, 10% experienced MACCEs within one year, and 2% had in-hospital MACCEs. Five of the original CHIP-PCI score variables emerged as independent predictors. including prior myocardial infarction, left ventricular ejection fraction (LVEF) < 30%, chronic kidney disease, left main coronary artery PCI, and non-radial access. Additionally, diabetes mellitus, anemia, and severe coronary calcification were identified as significant predictors of MACCEs. Incorporating these additional variables improved the score’s discriminatory value for predicting both one-year and in-hospital MACCEs [83].

### 8.3. Implications for Clinical Decision-Making

The identification of high-risk patients remains critical not only for tailoring antiplatelet therapy duration but also for broader clinical decision-making. Balancing the risks of bleeding and ischemic events is essential when determining the appropriateness and futility of certain interventions, particularly in elderly and complex patients. Tools like the CHIP-PCI score, enhanced with additional predictive factors, may help refine these assessments and optimize outcomes in this vulnerable population [26,74].

## 9. Conclusions

Bleeding risk remains a critical concern for elderly patients undergoing PCI, as they are disproportionately affected due to age-related physiological changes, comorbidities, and the complex interplay of antithrombotic therapies. Despite advancements in risk stratification tools such as the PRECISE-DAPT and ARC-HBR scores, their predictive accuracy in elderly populations remains suboptimal. Emerging tools like simplified clinical evaluation scores show promise in addressing these limitations by providing more tailored risk assessments. Optimizing the balance between minimizing bleeding complications and preventing ischemic events is essential to improving outcomes in this high-risk group, underscoring the need for individualized treatment strategies.

## Figures and Tables

**Table 1:** Definitions of bleeding events.

	Major Bleeding	Minor Bleeding
TIMI [41]	Any intracranial bleeding; clinically overt hemorrhage associated with a drop in hemoglobin of 5 g/dL; fatal bleeding (results in death < 7 days)	Mild bleeding that does not meet severe criteria
GUSTO [42]	Severe or life-threatening Intracerebral hemorrhage Resulting in substantial hemodynamic compromise requiring treatment Moderate Requiring blood transfusion but not resulting in hemodynamic compromise	
GRACE [45]	Requiring a transfusion of >2 units blood Resulting in a decrease in hematocrit of >10% Intracerebral hemorrhage Resulting in stroke or death	
BARC [43,44]	**Type 0:** No bleeding **Type 1:** Bleeding that is not actionable **Type 2:** Any actionable sign of hemorrhage not type 3, 4, or 5 but at least one (1) requiring non-surgical medical intervention by a healthcare professional, (2) leading to hospitalization or increased level of care, or (3) prompting evaluation **Type 3a:** Overt bleeding plus hemoglobin drop of 3 to <5 g/dL (provided hemoglobin drop is related to bleeding); any transfusion with overt bleeding **Type 3b:** Overt bleeding plus hemoglobin drop >5 g/dL (provided hemoglobin drop is related to bleeding) Cardiac tamponade Bleeding requiring surgical intervention for control Bleeding requiring intravenous vasoactive agents **Type 3c:** Intracranial hemorrhage; subcategories confirmed by autopsy or imaging or lumbar puncture Intraocular bleed compromising vision **Type 4:** Coronary artery bypass graft-related bleeding **Type 5:** Fatal bleeding	
*Continuation*	Major bleeding	Minor bleeding
ISTH [46]	**1** Fatal bleeding **2** Symptomatic bleeding in a critical area or organ, such as intracranial, intraspinal, intraocular, retroperitoneal, intra-articular or pericardial, or intramuscular with compartment syndrome **3** Bleeding causing a fall in hemoglobin level of 20 g/L (1.24 mmol L) or more or leading to transfusion of two or more units of whole blood or red cells	

**Table 2:** Data on elderly patients in pivotal trials of antithrombotic therapy in ACS.

Study	Population	Elderly (%)	Bleeding (Overall)	Bleeding (Elderly)
GUSTO-V [57]	STEMI (fibrinolysis)	Age ≥ 75: 13%	Abciximab: 4.6% No abciximab: 2.3% *p* < 0.001	Abciximab: 2.1% No abciximab: 1.1% *p* = 0.069
ISAR-REACT-2 [58]	PCI for NSTE-ACS	Age ≥ 70: 40%	Abciximab: 1.4% UFH: 1.4% *p* = NS	Abciximab: 2.7% UFH: 1.9% *p* = 0.46
ESPRIT [59]	PCI for NSTE-ACS	Age ≥ 65: NR	Eptifibatide: 1.0% Placebo: 0.4% *p* = 0.027	Not reported
PRISM-PLUS [60]	NSTE-ACS	Age ≥ 65: 49%	Tirofiban + heparin: 3.0% Heparin: 4.0% *p* = 0.34	Not reported
TRITON-TIMI 38 [48]	PCI for NSTE-ACS	Age ≥ 75: 13%	Prasugrel: 2.4% Clopidogrel: 1.8% *p* = 0.03	Not reported
CURE [49]	NSTE-ACS	Age ≥ 65: 49%	Clopidogrel: 3.7% Placebo: 2.7% *p* = 0.001	Not reported
COMMIT [61]	Acute MI	Age ≥ 70: 26%	Clopidogrel: 0.58% Placebo: 0.55% *p* = 0.59	Clopidogrel: 0.84%; placebo: 0.72% *p* = 1.48
CLARITY [62]	STEMI	Age ≥ 65: 29%	Clopidogrel: 1.9% Placebo: 1.7% *p* = 0.80	No increase in bleeding with clopidogrel by age
ACUITY [63]	PCI for NSTE-ACS	Age ≥ 75: 18%	Bivalirudin: 3.0% Heparin/GPI: 5.7% *p* < 0.001	Bivalirudin: 5.8% Heparin/GPI: 10.1% *p* < 0.05
CHAMPION-PCI [64]	PCI	Not reported	Cangrelor: 0.4% Clopidogrel: 0.3 *p* = 0.39	Not reported
PLATFORM [65]	PCI for NSTE-ACS	Age ≥ 75: 16%	Cangrelor: 0.2% Clopidogrel: 0.3% *p* = 0.17	Cangrelor: 2.1%; clopidogrel: 2.1% *p* = 0.94
PLATO [47]	PCI for STEMI/NSTE-ACS	Age ≥ 75: 15%	Ticagrelor: 11.6% Clopidogrel: 11.2% *p* = 0.043	Ticagrelor: 14.2%; clopidogrel: 13.3% *p* = NS

**Table 3:** PRECISE-DAPT variables.

Variable	Hazard Ratio	*p* Value	Assigned Score
Age (for each increase in 10 years)	1.34 (1.11–1.48)	0.005	0–19
Previous bleeding	4.14 (1.22–14.02)	0.023	0–26
White blood cell count (for each increase in 10^3^ cells per μL)	1.06 (0.99–1.13)	0.078	0–15
Hemoglobin at baseline (for each increase of 1 g/dL)	0.67 (0.53–0.84)	0.001	0–15
Creatinine clearance (for each increase of 10 mL/min)	0.90 (0.82–0.99)	0.004	0–25

**Table 4:** Validation of the PRECISE-DAPT score.

	TIMI Major and Minor Bleeding		TIMI Major Bleeding	
	C-Index (95% CI)	*p* Value *	C-Index (95% CI)	*p* Value *
Derivation Cohort	0.73 (0.61–0.85)	-	0.71 (0.57–0.85)	-
PLATO	0.70 (0.65–0.75)	0.06	0.68 (0.63–0.74)	0.01
BernPCI	0.66 (0.61–0.71)	0.09	0.65 (0.58–0.71)	0.17

* Compared to PARIS score as reference.

## Data Availability

Corresponding data may be made available upon reasonable request to the corresponding author.
